# A New Approach to the Assessment of Erectile Dysfunction Based on Vasomotion Monitored by the Flow-Mediated Skin Fluorescence (FMSF) Technique—A Preliminary Study

**DOI:** 10.3390/jcm13113210

**Published:** 2024-05-30

**Authors:** Jolanta Slowikowska-Hilczer, Renata Walczak-Jedrzejowska, Daria Adamczewska, Piotr Byczkiewicz, Katarzyna Marchlewska, Joanna Katarzynska, Jerzy Gebicki

**Affiliations:** 1 Department of Andrology and Reproductive Endocrinology, Medical University of Lodz, 92-213 Lodz, Poland; renata.walczak-jedrzejowska@umed.lodz.pl (R.W.-J.); daria.adamczewska@umed.lodz.pl (D.A.); piotr.byczkiewicz@umed.lodz.pl (P.B.); katarzyna.marchlewska@umed.lodz.pl (K.M.); 2 Angionica Ltd., 90-924 Lodz, Poland; joanna.katarzynska@angionica.com.pl (J.K.); jerzy.gebicki@angionica.com.pl (J.G.); 3 Institute of Applied Radiation Chemistry, Lodz University of Technology, 90-924 Lodz, Poland

**Keywords:** microcirculation, vasomotion, testosterone, erectile dysfunction

## Abstract

**Background:** Erectile dysfunction (ED) most often has vascular etiology and usually is the earliest symptom of vascular dysfunction. The aim of this study was to evaluate vascular dysfunction with the use of the Flow-Mediated Skin Fluorescence (FMSF) technique in men with and without ED. **Methods:** Included were 39 men (median age 53) with ED and 40 men (median age 41.5) without ED. Medical interview, physical examination, and anthropometrical measurements were performed for all participants. The serum total testosterone, LH, and SHBG determinations were performed in patients with ED, and the Free Testosterone Index (FTI) was calculated. The FMSF technique was used to measure the microcirculatory oscillations at the baseline and to determine the flowmotion (FM) and vasomotion (VM) parameters. The Normoxia Oscillatory Index (NOI) was calculated, which represents the contribution of the endothelial (ENDO) and neurogenic (NEURO) oscillations relative to all oscillations detected at low-frequency intervals (<0.15 Hz): NOI = (ENDO + NEURO)/(ENDO + NEURO + VM). **Results:** In men with ED were found significantly lower FM and VM parameters, but the NOI was significantly higher in comparison to men without ED. VM and FM correlated significantly positively with erectile function, orgasmic function, and general sexual satisfaction in the whole group and the FTI in the ED group. The thresholds of 53.5 FM (AUC = 0.7) and 8.4 VM (AUC = 0.7) were predictive values for discriminating men with ED. **Conclusions:** It was shown that the FMSF diagnostic technique may be helpful in the early diagnosis of microcirculation dysfunction due to impaired vasomotion caused by decreased testosterone activity.

## 1. Introduction

An erection is a neurovascular event that consists of a vascular phase that is the consequence of a balance between arterial inflow and venous outflow. The cavernosal arteries supply blood to the corpora cavernosa of the penis. During erection, relaxation of the trabecular smooth muscle results in increased blood flow to the corpora cavernosa. Distension of the sinuses in the corpora cavernosa causes mechanical compression of the emissary veins in the tunica albuginea, which impedes their ability to drain blood and thereby results in penile rigidity [1,2].

It is known that androgens play a modulating role by their effect on the libido and the frequency of sexual activity [3]. However, some recent studies have documented a direct role of testosterone on cavernous smooth muscle cells, involving NO, RHO-associated protein kinase (ROCK), phosphodiesterase 5 (PDE5), and the adrenergic response [4].

Erectile dysfunction (ED) is related to several comorbidities, such as vascular disorders (atherosclerosis and failure of the corporal veno-occlusive mechanism); neurogenic disorders (diabetic neuropathy); psychiatric disorders (depression); endocrine disorders (hypogonadism and hyperprolactinemia); systemic diseases (cardiovascular, renal, and hepatic); drug-induced; and some genitourinary diseases related to surgery [5]. Among the different pathogenic mechanisms of ED, vascular etiology is the most common cause [6,7]. Increasing evidence suggests an association between ED and cardiovascular diseases (CVDs), with an increased prevalence of ED in cardiovascular patients and an increased prevalence of CVD in patients with ED [8,9]. Among the clinical manifestations of atherosclerotic disease, ED usually proceeds the onset of coronary diseases by approximately 5 years [10,11]. Smaller diameters of penile arteries (1–2 mm) suffer earlier from atherosclerotic plaque burden, leading to arterial obstruction and flow compromise. In turn, larger coronary (3–4 mm) or carotid arteries (5–7 mm) are affected later.

It has also been demonstrated that low testosterone levels are associated with major adverse cardiovascular events (MACEs) and a higher lethality of MACEs in patients with ED [12,13]. Moreover, it has been revealed that, in hypogonadal men with a history of CVD, testosterone replacement therapy improves and preserves erectile function over prolonged periods with concurrent sustained improvements in cardiometabolic risk factors [14]. Thus, men with ED should be screened for comorbidities. It is a chance for them to improve not only their sexual but also their overall health.

It has been documented based on rodent models that testosterone can affect spontaneous rhythmical variations in testicular arteriolar blood flow known as vasomotion (VM) [15,16]. VM is caused by spontaneous myogenic activity in arterioles, and impaired VM can lead to vascular resistance. The testosterone effect on VM can be manifested by an increased amplitude of myogenic microcirculatory oscillations. An important hypothesis can be formulated on whether a testosterone effect on VM can also be seen in systemic peripheral microcirculation. Such a hypothesis is worth careful verification, as it may turn out that testosterone deficiency can be linked to impaired systemic myogenic microcirculatory oscillations resulting in early microvascular dysfunction.

A new diagnostic technique named Flow-Mediated Skin Fluorescence (FMSF), which enables a noninvasive evaluation of the vascular circulation and metabolic regulation, was introduced in 2017 [17,18,19,20,21]. The FMSF technique is based on monitoring the intensity of nicotinamide adenine dinucleotide (NADH) fluorescence from skin tissue on the forearm in response to blocking and releasing blood flow as a function of time. It has been shown that oscillations in the microcirculation, known as flowmotion (FM), can be successfully monitored by the FMSF technique and used for characterization of the microcirculatory status [19,22]. The FM intensity observed in the low-frequency domain (below 0.15 Hz) contains VM representing myogenic oscillations observed in the frequency range of 0.052–0.15 Hz. Thus, the FMSF technique allows for precise analysis of both the FM and VM intensities.

The aim of this study is to present a new approach to the assessment of microcirculation dysfunction in men with ED based on quantitative measurements of the FM and VM intensities using the FMSF technique. Early dysfunction of the microcirculation caused by impaired testosterone activity will be discussed. Both patophysiological and diagnostic aspects of this preliminary observation will be considered.

## 2. Materials and Methods

### 2.1. Participants

Approval for the study was obtained from the management of the Central Hospital of the Medical University of Lodz (07/2021). All participants received a detailed description of the study and signed the informed consent.

The inclusion criteria for participation in the study were erectile dysfunction, age 18–80 years, and conscious agreement. The exclusion criteria were age <18 and >80 years and disagreement to participate in the study. The enrollment was performed among patients diagnosed in the Andrology and Reproductive Endocrinology Outpatients Clinic in the Central Hospital of Medical University of Lodz and among men from the general population.

All participants had a medical interview and physical examination by an andrologist.

### 2.2. Anthropometric Measurements

Body weight was measured to the nearest 0.1 kg using an electronic scale (SECA UK Ltd., Birmingham, UK), height to the nearest 1 mm using a stadiometer (Leicester Height Measure, SECA UK Ltd.), and the body circumferences (waist and hip) using anthropometric tape. Then, the body mass index (BMI, weight/height^2^) and waist-to-hip ratio (WHR) were calculated. The fat mass was measured using an electrical bioimpedance analysis machine (Body Composition Analyzer TBF-300, Tanita, Europe B.V.), and then, the fat tissue percentage of the body mass was calculated. The blood pressure was measured using a semi-automated device (Omron M6 Comfort, HEM-7221-E).

### 2.3. Questionnaires

All participants completed the following questionnaires:

– International Index of Erectile Function 15 (IIEF-15)—standardized and validated 15-item self-evaluation scale that provides evaluations of erectile function, orgasmic function, sexual desire, satisfaction in sexual intercourse, and general satisfaction of sexual life. The score for each question was 0–5. Those men who did not have intercourse during last month because of a severe problem with their erection were also included in the study.
– Beck Depression Inventory (BDI)—a 21-question multiple-choice self-report inventory to measure the severity of depression: 0–11 points—lack of depression, 12–19—light depression, 20–25—moderate depression, and 26–63—severe depression.
– Androgen Deficiency in Aging Male (ADAM)—a 10-question self-report inventory to measure symptoms of hypogonadism.
– Aging Male Symptoms scale (AMS)—consists of 17 questions, each scored from 1 to 5.
– International Physical Activity Questionnaire (IPAQ)—a set of 4 questionnaires to assesses the types of intensity of physical activity and sitting time. Metabolic equivalents (METs) of physical activity and MET minutes/week were calculated [23].
– questionnaire that includes questions about general health information, such as current diagnosed diseases and pharmacological drug intake during the last 3 months, and lifestyle and diet, such as alcohol, coffee, energy drinks, illegal substances consumption, and smoking.

### 2.4. FMSF Measurements

The microcirculatory oscillations were measured with the use of AngioExpert device (Angionica Ltd.). The measurement protocol has been described in detail elsewhere [19]. The AngioExpert device uses the Flow-Mediated Skin Fluorescence (FMSF) technique, which measures changes in the intensity of NADH fluorescence from the skin on the forearm in response to blocking (following cuff occlusion) and releasing blood flow. The skin is the largest organ of the human body and is characterized by specific metabolism. The epidermal layer of the skin is not directly vascularized, and oxygen and nutrients are transported from the dermis by diffusion. Therefore, epidermal cell metabolism can be considered a unique and sensitive marker of early dysfunction in vascular circulation and metabolic regulation. Oscillations in the microcirculation (FM) are a well-recognized characteristic of cutaneous blood flow. Two different periods of oscillations can be distinguished in the FMSF signal: basal oscillations at rest and during the reperfusion following post-occlusive reactive hyperemia (PORH). The blood flow oscillations in the low-frequency range (<0.15 Hz) fit into several periodic activities, classified as endothelial (<0.021 Hz), neurogenic (0.021–0.52 Hz), and myogenic (MYO = VM) (0.052–0.15 Hz). The frequencies of the oscillations contained in the FMSF signal were analyzed using the Fast Fourier-Transform (FFT) algorithm. Periodograms were derived from the FFT of the signal, with rectangular windowing and the Power Spectra Density (PSD) calculated as a mean squared amplitude.

In this preliminary study, only the microcirculatory oscillations measured at the baseline are analyzed, and the FM and VM intensities are determined. Additionally, the NOI (Normoxia Oscillatory Index) parameter is calculated. The NOI parameter represents the contribution of endothelial (ENDO) and neurogenic (NEURO) oscillations relative to all oscillations detected at low-frequency intervals (<0.15 Hz) [24,25]. The formula for the NOI calculation: NOI = (ENDO + NEURO)/(ENDO + NEURO + VM).

### 2.5. Laboratory Determinations

Men with ED had laboratory determinations of total testosterone (T), luteinizing hormone (LH), and sex hormone-binding globulin (SHBG) in the same hospital laboratory. Fasting blood was taken in the morning hours (8:00–10:00). The serum levels of LH and total testosterone were determined using enhanced chemiluminescence on a VITROS ECi Immunodiagnostic System with MicroWell technology (Ortho-Clinical Diagnostics, Bridgend, UK). The intra- and inter-assay coefficients of variations (CVs) were <3.8% and <7.4% for the measurements of testosterone and <8.8% and <12.8% for LH. The levels of SHBG were determined by the chemiluminescent microparticles immunoassay on the ARCHITECT System (Abbott Laboratories, Green Oaks, IL, USA). The intra- and inter-assay CVs for SHBG were <5.2% and <10.0%, respectively. The Free Testosterone Index (FTI, total testosterone/SHBG × 100) was calculated.

The results of the hormonal determinations from men with ED were compared with the results obtained from our previous study from a group of 80 men from the general population aged 20–39 years [26,27,28].

### 2.6. Statistical Analysis

All statistical analyses were performed using Statistica for Windows software, version 13.3 (StatSoft Polska on the license of the Medical University in Lodz). The normality of the data distribution was analyzed using the Shapiro–Wilk test. As the results were non-normally distributed, the descriptive statistics were presented using the median and interquartile range (25–75 percentiles) for continuous variables or frequencies (percentages) for categorical variables. Differences between two or three groups were tested using the Mann–Whitney *U* test or analysis of variance (ANOVA) and post hoc comparisons for the Kruskal–Wallis test for continuous variables, respectively, and weighted prevalence comparisons were conducted using the Chi^2^ test for categorical variables. The receiver operating characteristic (ROC) curve and the area under the curve (AUC) were applied to calculate the thresholds of FM and VM to discriminate men with ED from men without ED. The ROC analysis took into account the standard error (SE), 95% confidence interval (95% CI), sensitivity, and specificity. The predictive values of the AUCs were characterized as follows: 0.9–1.0 as excellent predictive value, >0.8–0.9 as good predictive value, >0.7–0.8 as satisfactory predictive value, >0.6–0.7 as moderate predictive value, and 0.5–0.6 as insufficient predictive value. Spearman’s rank correlations were conducted between results of the FMSF parameters and other studied parameters. A *p*-value <0.05 was considered statistically significant.

## 3. Results

Included in the study were 39 men (age 27–72, median 53 years) with ED and 40 men (age 24–57, median 41.5 years) without ED (control group).

Men with ED more often reported current diseases in comparison to men without ED (82% vs. 40%, *p* < 0.001). As comorbidities, they reported CVD (40%) and diabetes (17%) the most frequently. Men without ED reported CVD (22%) and diabetes (3%) significantly less often, *p* < 0.001. Men with ED more frequently took medicines (77% vs. 38%, *p* < 0.01).

Stimulants were used more often by men without ED in comparison to those with ED: energetics 35% vs. 9% (*p* < 0.05), drugs 7.5% vs. 2.5% (*p* < 0.01), nicotine 35% vs. 26% (*p* < 0.01), and androgen-anabolic steroids (10% vs. 0%). Heavy alcohol drinking (>75 g/day) was reported more often by men with ED (2.5% vs. 0%), as well as the level of frequent coffee consumption (>3 cups/day) (25.5% vs. 22.5%); however, this was not statistically significant.

Table 1 shows comparisons between men with and without ED. Men with ED were significantly older. They had significantly higher waist circumferences and WHR. The results of the IIEF-15 questionnaire showed, besides worse erectile function, also significantly worse orgasmic function, sexual desire, satisfaction in sexual intercourse, and general satisfaction of sexual life. Moreover, they had higher BDI scores, which indicates a worse mood. They also had significantly higher scores in the ADAM and AMS questionnaires, indicating more symptoms of androgen deficiency and aging. Among the patients with ED, 49% had serum testosterone levels below the reference value—3.5 ng/mL [29]. They also had low FTI values.

**Figure 1:**
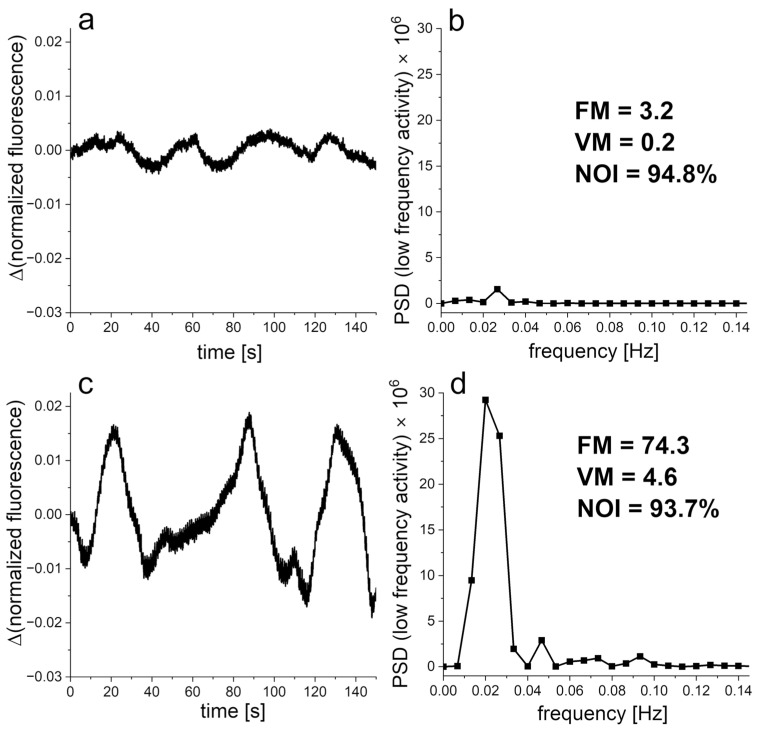

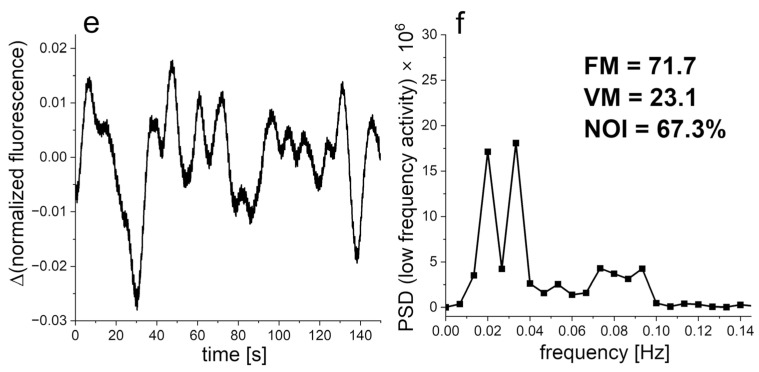
Exemplary of Flow-Mediated Skin Fluorescence (FMSF) baseline traces recorded for (**a**,**b**) a patient with ED (age 42 y), (**c**,**d**) a patient with ED (age 37 y), and (**e**,**f**) a patient without ED (age 34 y). Changes in the fluorescence signal relative to the normalized baseline (**left**) and the corresponding Power Spectral Density (PSD) of the fluorescence signal in the intervals of endothelial (<0.021 Hz), neurogenic (0.021–0.052 Hz), and myogenic (0.052–0.15 Hz) activity (**right**). FM—flowmotion; NOI—Normoxia Oscillatory Index, and VM—vasomotion.

The low-frequency microcirculation oscillations at the baseline measured by the FMSF technique showed significantly lower values of the FM and VM parameters in men with ED. However, the NOI parameter was significantly higher in comparison to men without ED. Exemplary FMSF baseline traces recorded for two patients with ED and one patient without ED are presented in Figure 1. It is clearly seen that the VM parameters representing myogenic microcirculatory oscillations are much lower in two patients with ED and the NOI parameters are appropriately higher, supporting the results presented.

Table 2 shows that, in the whole group of men, the VM and FM parameters correlated significantly negatively with age, waist circumference, WHR, and fat mass.

Correlations between the results of IIEF-15 in the whole group of men presented in Table 2 have shown that VM and FM correlated significantly positively with erectile function, orgasmic function, and general sexual satisfaction. Correlations between the results of hormonal determinations and the results of FMSF measurements in the group with ED revealed that the FTI correlated significantly positively with FM (r = 0.51) and VM (r = 0.46).

**Figure 2:**
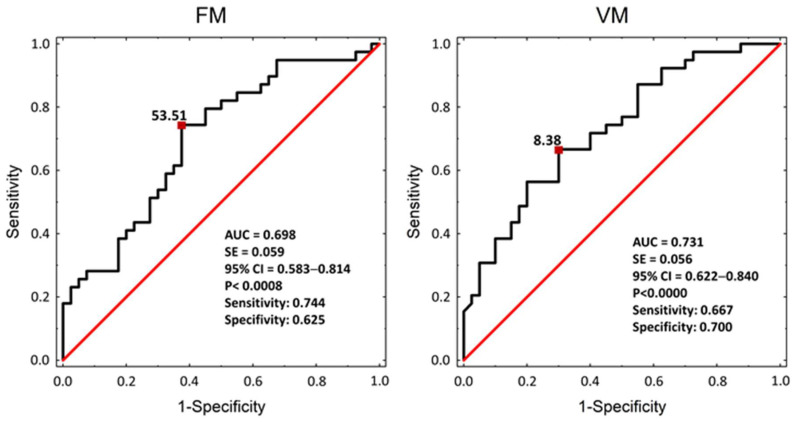
Receiver operating characteristic (ROC) curve and cut-off points for flowmotion (FM) and vasomotion (VM) to discriminate men with ED from men without ED. Abbreviations: AUC—area under the curve, CI—confidence interval, SE—standard error, and *p*—statistical significance between the obtained AUC vs. AUC = 0.500 (red line); statistical significance was reached when *p* < 0.05. The following levels of the AUC were presumed: >0.6–0.7—moderate predictive value and >0.7–0.8—satisfactory predictive value.

The receiver operating characteristic (ROC) curves for the VM and FM parameters discriminating men with ED from men without ED are presented in Figure 2. The FM threshold of 53.5 (AUC = 0.7) had a moderate predictive value, and the threshold of 8.4 VM (AUC = 0.7) had a satisfactory predictive value.

## 4. Discussion

It has already been proven that ED is more common in elderly men [30]. In our study, we confirmed that ED is seen more often in men above 40, who reported more symptoms of aging in the AMS and hypogonadism in the ADAM questionnaire, as well as more comorbidities, with CVD and diabetes as the most common. The men with ED had significantly increased waist circumferences and WHR, anthropometric parameters that were found to be risk factors for CVD [31,32].

In addition to poor erectile function, these men showed worse results in orgasmic function, sexual desire, satisfaction in sexual intercourse, and general satisfaction of sexual life. These findings may be related to the relatively low mean testosterone serum levels. It is not surprising, because the mean age in this group was 53 years. The European Male Aging Study (EMAS) results revealed that around 30% of 40–79-year-old men reported ED, and 6% reported severe orgasmic impairment, both of which were closely associated with age and concomitant morbidities [33]. The EMAS results also showed that the total testosterone serum levels decreased 0.4% per year and free testosterone levels 1.3% per year after the age of 40 [34]. Moreover, an inverse relationship between an increasing number of sexual symptoms and a decreasing testosterone level was observed. In our previous study, we revealed in 80 Polish men aged 20–39 years that the mean serum testosterone level was about 5 ng/mL [26,27,28], thus significantly higher in comparison to the men aged 40–72 years presented in this study.

Moreover, in the group of patients with ED, higher BDI scores were found in comparison to men without ED. In the whole group of the studied men, an erectile function score correlated significantly negatively with the BDI score. These results most likely point to a psychic component as the additional reason for ED. It is known that one of the symptoms of depression is poor sexual function [35].

Nowadays, penile Doppler sonography is an essential tool for differentiating between vascular and nonvascular causes of ED. This ultrasound technology produces a detailed description of the hemodynamics of the patient’s erection cycle [36]. However, it is minimally invasive when assisted by intracavernosal injections such as alprostadil. Moreover, it is criticized because of marked protocol heterogeneity, rendering data interpretation a problem [37]. The FMSF diagnostic technique is a relatively new noninvasive and easy-to-use method to evaluate the vascular circulation [20] that may be helpful in screening for vascular problems. In this preliminary study, it was used for the first time to evaluate the microcirculation in men with ED. With the use of this device, we were able to show that some of the FMSF parameters, particularly related to microcirculation oscillations observed at the baseline, may be helpful in diagnosing problems of vascular circulation linked to ED. The parameters such as VM and FM were significantly lower, and the NOI parameter was significantly higher in men with ED. The VM parameter seems to carry a powerful potential to be used for diagnostic purposes. The VM parameter characterizes the intensity of myogenic microcirculatory oscillations directly responsible for vascular resistance [15,16]. It was observed that microcirculatory function decreases gradually with age [38]; however, in some circumstances, it may be found in younger men [24].

We have shown that both the VM and FM parameters significantly correlate with FTI, which suggests a direct involvement of free (biologically active) testosterone in the regulation of vascular resistance. The lower level of the FTI in men with ED translates to the lower intensity of microvascular oscillations at low-frequencies regions (<0.15 Hz). This new observation is particularly important, as it places impaired testosterone regulations as a key factor responsible for vascular resistance. It may turn out that such impaired testosterone regulation can be a critical factor affecting vascular circulation, leading to a predisposition of men toward ED and cardiovascular diseases.

Previously, it has been shown using the FMSF technique that psychological stress affects microcirculation oscillations [25]. Psychological stress is associated with increased levels of norepinephrine in the circulation, which can cause microvascular vasoconstriction. In the case of healthy individuals, such vasoconstriction can be compensated for by an increased activity of myogenic microcirculatory oscillations measured by the VM parameter, with an adequate decrease of the NOI parameter. Chronic psychological stress can lead to the development of serious vascular circulatory disorders. As it was shown above, men with ED have impaired vasomotion manifested by lower values of the VM parameter, and vasoconstriction due to psychological stress may not be effectively compensated for by an increased activity of myogenic oscillations (vasomotion). Also, depression present among men with ED can often lead to vascular dysfunction due to vascular resistance caused by impaired testosterone function.

The strength of the study is a novel observation linking low free testosterone activity with microvascular dysfunction and the risk of ED. It can be hypothesized that that the vascular resistance caused by impaired testosterone function is an early step of microcirculatory dysfunction, which can further develop into a more pronounced vascular dysfunction involving macrocirculation.

The limitation of the study is the low number of studied men with and without ED. However, it was a preliminary study to check if the FMSF technique may be helpful in the diagnosis of vascular problems in men with ED. Moreover, in this study, the results of the FMSF technique with PORH provocation are not shown, which are particularly useful to evaluate macrocirculation. Comparing the results of FMSF and the penile Doppler sonography also seems important. Such studies on more numerous groups with broader hormonal research are planned by us.

## 5. Conclusions

In this preliminary study, it was shown that the FMSF diagnostic technique may be helpful to diagnose microcirculation dysfunction due to impaired vasomotion (myogenic oscillations). The VM parameter may be considered as a promising candidate for such a test. Moreover, it seems that there might be a relationship between low VM values and decreased testosterone activity in men with ED. However, a study on a larger number of patients to confirm these findings is needed.

The use of the FMSF technique is particularly attractive due to its noninvasive nature. It can be useful for screening of the male population for microvascular disorders, possibly by the use of electronic wearable devices. The early diagnosis of microvascular problems can encourage men, even below 40, to start changes in their lifestyle (increasing physical activity and lowering body fat mass) to prevent erectile dysfunction, cardiovascular disease, and, finally, major adverse cardiovascular events.

## Figures and Tables

**Table 1:** Comparisons between men with and without erectile dysfunction (ED).

Parameters	Men without ED N = 40 Median (Q1–Q3)	Men with ED N = 39 Median (Q1–Q3)
*Age (years)*	41.5 (33.5–51.5)	53 (48–63) ^3^
***Anthropometric measurements***		
Weight (kg)	86 (80.5–104.5)	92 (83–100)
BMI (kg/m^2^)	27 (22–38)	29 (26–32)
Waist circumference (cm)	94.5 (90.8–104)	102.6 (95.3–110) ^2^
WHR	0.92 (0.88–0.98)	0.98 (0.94–1) ^1^
Fat mass (%)	23.2 (20.2–27.2)	26.0 (22.2–26.9)
BP systolic	139 (129.5–147.5)	136 (124–149)
BP diastolic	85.5 (76.5–93.5)	83 (77–91)
***Questionnaires***		
IIEF-15		
– erectile function	29 (28–30)	17 (9–22) ^3^
– orgasmic function	10 (10–10)	7 (2–10) ^3^
– sexual desire	7 (6–8.5)	6 (5–7) ^3^
– satisfaction in sexual intercourse	13 (11–14)	8 (3–10) ^3^
– general satisfaction	9 (8–10)	6 (3–10) ^3^
BDI (score)	4 (1–8)	8 (5–10) ^3^
ADAM (Yes)	3.5 (2–5)	5 (3–7) ^1^
AMS (score)	28.5 (23–34)	36 (28–42) ^3^
IPAQ (MET-min/week)	3258 (1224–5370)	2772 (1356–5493)
***FMSF measurements***		
FM	65.7 (31.2–137.2)	33.9 (16.6–57.5) ^2^
VM	14.3 (6.9–34.2)	4.9 (1.6–14.8) ^3^
NOI (%)	76.4 (63.1–85.5)	81.9 (71–92.2)^1^
***Laboratory determinations***		
T total (ng/mL)	5.3 ± 1.6 (2.1–9.9) *	3.1 (2–4.6)
SHBG (nmol/l)	25.9 ± 10.6 (10.7–59.5) *	39.3 (26.4–61.5)
FTI	78.1 ± 29.1 (26.8–167.3) *	31.7 (26.4–61.5)
LH (U/L)	4.6 ± 1.8 (0.8–8.4) *	3 (2.4–3.6)

^1^ *p* < 0.05, ^2^ *p* < 0.01, and ^3^ *p* < 0.001; Q—quartile; * adopted from Slowikowska-Hilczer [28] (mean ± SD). Abbreviations: ADAM—Androgen Deficiency in Aging Male, AMS—Aging Male Symptoms scale, BDI—Beck Depression Inventory, BMI—body mass index, BP—blood pressure, FM—flowmotion, FMSF—Flow-Mediated Skin Fluorescence, FTI—Free Testosterone Index, IIEF—International Index of Erectile Function, IPAQ—International Physical Activity Questionnaire, LH—luteinizing hormone, MET—metabolic equivalent, NOI—Normoxia Oscillatory Index, SHBG—sex hormone-binding globulin, T—testosterone, VM—vasomotion, WHR—waist-to-hip ratio.

**Table 2:** Spearman r correlations (r_s_) between the results of anthropometric measurements, results of questionnaires, and FMSF parameters (flowmotion: FM and vasomotion: VM) in the whole group of participants (N = 79).

Parameters	FM r_s_	VM r_s_
***Anthropometric measurements***		
Age	−0.44 ^3^	−0.52 ^3^
Weight	−0.18	−0.14
Height	0.08	0.08
BMI	−0.24 ^2^	−0.21
Waist circumference	−0.33 ^2^	−0.29 ^2^
WHR	−0.29 ^2^	−0.33 ^2^
Fat mass	−0.39 ^3^	−0.38 ^3^
BP systolic	0.08	−0.3
BP diastolic	0.05	0.00
***Questionnaires***		
Erectile function	0.34 ^2^	0.46 ^3^
Orgasmic function	0.24 ^1^	0.34 ^2^
Sexual desire	0.11	0.24 ^1^
Satisfaction in sexual intercourse	0.22	0.30 ^2^
General satisfaction	0.25 ^1^	0.38 ^3^
BDI	−0.15	−0.30 ^2^
ADAM	−0.04	−0.22 ^1^
AMS	−0.20	−0.32 ^2^
IPAQ (MET-min/week)	−0.05	−0.06

^1^ *p* < 0.05, ^2^ *p* < 0.01, and ^3^ *p* < 0.001; Statistical significance in Spearman’s rank correlation was reached when *p* < 0.05. The interpretation of the r_s_ values: <0.2 is a lack of linear dependence, 0.2–0.4 is a weak dependence, and >0.4–0.7 is a moderate dependence.

## Data Availability

Dataset is available on request from the authors.
